# Matching health information seekers' queries to medical terms

**DOI:** 10.1186/1471-2105-13-S14-S11

**Published:** 2012-09-07

**Authors:** Lina F Soualmia, Elise Prieur-Gaston, Zied Moalla, Thierry Lecroq, Stéfan J Darmoni

**Affiliations:** 1 LIM & Bio EA 3969, Université Paris XIII, Sorbonne Paris Cité, 93017 Bobigny, France; 2 LITIS-TIBS EA 4108 & CISMeF Rouen University Hospital, 76031 Rouen, France

## Abstract

**Background:**

The Internet is a major source of health information but most seekers are not familiar with medical vocabularies. Hence, their searches fail due to bad query formulation. Several methods have been proposed to improve information retrieval: query expansion, syntactic and semantic techniques or knowledge-based methods. However, it would be useful to clean those queries which are misspelled. In this paper, we propose a simple yet efficient method in order to correct misspellings of queries submitted by health information seekers to a medical online search tool.

**Methods:**

In addition to query normalizations and exact phonetic term matching, we tested two approximate string comparators: the similarity score function of Stoilos and the normalized Levenshtein edit distance. We propose here to combine them to increase the number of matched medical terms in French. We first took a sample of query logs to determine the thresholds and processing times. In the second run, at a greater scale we tested different combinations of query normalizations before or after misspelling correction with the retained thresholds in the first run.

**Results:**

According to the total number of suggestions (around 163, the number of the first sample of queries), at a threshold comparator score of 0.3, the normalized Levenshtein edit distance gave the highest F-Measure (88.15%) and at a threshold comparator score of 0.7, the Stoilos function gave the highest F-Measure (84.31%). By combining Levenshtein and Stoilos, the highest F-Measure (80.28%) is obtained with 0.2 and 0.7 thresholds respectively. However, queries are composed by several terms that may be combination of medical terms. The process of query normalization and segmentation is thus required. The highest F-Measure (64.18%) is obtained when this process is realized before spelling-correction.

**Conclusions:**

Despite the widely known high performance of the normalized edit distance of Levenshtein, we show in this paper that its combination with the Stoilos algorithm improved the results for misspelling correction of user queries. Accuracy is improved by combining spelling, phoneme-based information and string normalizations and segmentations into medical terms. These encouraging results have enabled the integration of this method into two projects funded by the French National Research Agency-Technologies for Health Care. The first aims to facilitate the coding process of clinical free texts contained in Electronic Health Records and discharge summaries, whereas the second aims at improving information retrieval through Electronic Health Records.

## Background

The Internet is fast becoming a recognized source of information in many fields, including health. In this domain, as in others, users are now experiencing huge difficulties in finding precisely what they are looking for among the numerous documents available online, and this in spite of existing tools. In medicine and health-related information accessible on the Internet, general search engines, such as Google, or general catalogues, such as Yahoo, cannot solve this problem efficiently [1]. This is because they usually offer a selection of documents that turn out to be either too large or ill-suited to the query. Free text word-based search engines typically return innumerable completely irrelevant hits, which require much manual weeding by the user, and also miss important information resources.

In this context, several health gateways [2] have been developed to support systematic resource discovery and help users find the health information they are looking for. These information seekers may be patients but also health professionals, such as physicians searching for clinical trials. Health gateways rely on thesauri and controlled vocabularies. Some of them are evaluated in [3]. Thesauri are a proven key technology for effective access to information since they provide a controlled vocabulary for indexing information. They therefore help to overcome some of the problems of free-text search by relating and grouping relevant terms in a specific domain. Nonetheless, medical vocabularies are difficult to handle by non-professionals.

Many tools have been developed to improve information retrieval from such gateways. They exploit techniques such as natural language processing, statistics, lexical and background knowledge ... *etc*. However, a simple spelling corrector, such as Google's "*Did you mean:*" or Yahoo's "*Also try:*" feature may be a valuable tool for non-professional users who may approach the medical domain in a more general way [4]. Such features can improve the performance of these tools and provide the user with the necessary help. In fact, the problem of spelling errors represents a major challenge for an information retrieval system. If the queries (composed by one or multiple words) generated by information seekers remain undetected, this can result in a lack of outcome in terms of search and retrieval. A spelling corrector may be classified in two categories. The first relies on a dictionary of well-spelled terms and selects the top candidate based on a string edit distance calculus. An approximate string matching algorithm, or a function, is required to detect errors in users' queries. It then recommends a list of terms from the dictionary that are similar to each query word. The second category of spelling correctors uses lexical disambiguation tools in order to refine the ranking of the candidate terms that might be a correction of the misspelled query. Several studies have been published on this subject. We cite the work of Grannis [5] which describes a method for calculating similarity in order to improve medical record linkage. This method uses different algorithms such as Jaro-Winkler, Levenshtein [6] and the longest common subsequence (LCS). In [7] the authors suggest improving the algorithm for computing Levenshtein similarity by using the frequency and length of strings. In [8] a phonetic transcription corrects users' queries when they are misspelled but have similar pronunciation (*e.g*. Alzaymer vs. Alzheimer). In [9] the authors propose a simple and flexible spell checker using efficient associative matching in a neural system and also compare their method with other commonly used spell checkers.

In fact, the problem of automatic spell checking is not new. Indeed, research in this area started in the 1960's [10] and many different techniques for spell checking have been proposed since then. Some of these techniques exploit general spelling error tendencies and others exploit phonetic transcription of the misspelled term to find the correct term. The process of spell checking can generally be divided into three steps (i) *error detection*: the validity of a term in a language is verified and invalid terms are identified as spelling errors (ii) *error correction*: valid candidate terms from the dictionary are selected as corrections for the misspelled term and (iii) *ranking*: the selected corrections are sorted in decreasing order of their likelihood of being the intended term. Many studies have been performed to analyze the types and the tendencies of spelling errors for the English language. According to [11] spelling errors are generally divided into two types, (i) *typographic errors *and (ii) *cognitive errors*. *Typographic errors *occur when the correct spelling is known but the word is mistyped by mistake. These errors are mostly related to keyboard errors and therefore do not follow any linguistic criteria (58% of these errors involve adjacent keys [12] and occur because the wrong key is pressed, or two keys are pressed, or keys are pressed in the wrong order ... *etc*.). *Cognitive errors*, or orthographic errors, occur when the correct spelling of a term is not known. The pronunciation of the misspelled term is similar to the pronunciation of the intended correct term. In English, the role of the sound similarity of characters is a factor that often affects error tendencies [12]. However, phonetic errors are harder to correct because they deform the word more than a single insertion, deletion or substitution. Indeed, over 80% of errors fall into one of the following four single edit operation categories: (i) single letter insertion; (i) single letter deletion; (iii) single letter substitution and (iv) transposition of two adjacent letters [10,11].

The third step in spell-checking is the ranking of the selected corrections. Main spell-checking techniques do not provide any explicit mechanism. However, statistical techniques provide ranking of the corrections based on probability scores with good results [13-15].

HONselect [16] is a multilingual and intelligent search tool integrating heterogeneous web resources in health. In the medical domain, spell-checking is performed on the basis of a medical thesaurus by offering information seekers several medical terms, ranging from one to four differences related to the original query. Exploiting the frequency of a given term in the medical domain can also significantly improve spelling correction [17] : edit distance technique is used for correction along with term frequencies for ranking. In [18] the authors use normalization techniques, aggressive reformatting and abbreviation expansion for unrecognized words as well as spelling correction to find the closest drug names within RxNorm for drug name variants that can be found in local drug formularies. It returns only drug name suggestions. To match queries with the MeSH thesaurus, Wilbur *et al. *[19] propose a technique on the noisy channel model and statistics from the PubMed logs.

Research has focused on several different areas, from pattern matching algorithms and dictionary searching techniques to optical character recognition of spelling corrections in different domains. However, relatively few groups have studied spelling corrections regarding medical queries in French. In this paper, a simple method is proposed : it combines two approximate string comparators, the well-known Levenshtein [6] edit distance and the Stoilos function similarity defined in [20] for ontologies. We apply and evaluate these two distances, alone and combined, on a set of sample queries in French submitted to the health gateway CISMeF [21]. The queries may be submitted both by health professionals in their clinical practice as well as patients. The system we have designed aims to correct errors resulting in non-existent terms, and thus reducing the silence of the associated search tool.

## Methods

### Similarity functions

Similarity functions between two text strings *S_1 _*and *S_2 _*give a similarity or dissimilarity score between *S_1 _*and *S_2 _*for approximate matching or comparison. For example, the strings "*Asthma*" and "*Asthmatic*" can be considered similar to a certain degree. Modern spell-checking tools are based on the simple Levenshtein edit distance [6] which is the most widely known. This function operates between two input strings and returns a score equivalent to the number of substitutions and deletions needed in order to transform one input string into another. It is defined as the minimum number of elementary operations that is required to pass from a string *S_1 _*to a string *S_2_*. There are three possible transactions: replacing a character with another, deleting a character and adding a character. This measure takes its values in the interval [0, ∞]. The Normalized Levenshtein [22] (*LevNorm*) in the range [0, 1] is obtained by dividing the distance of Levenshtein *Lev(S_1_*, *S_2_) *by the size of the longest string and it is defined by the following equation (1):

(1) $$LevNorm\left(S_{1},S_{2}\right)=\frac{Lev\left(S_{1},S_{2}\right)}{Max\left(\left|S_{1}|,|S_{2}\right|\right)}$$

*LevNorm (S_1_, S_2_) *∈ [0, 1] as *Lev(S_1_, S_2_) *<*Max*(|*S_1_*|,|*S_2_*|).

For example, *LevNorm(eutanasia*, *euthanasia) *= 0.1, as *Lev(eutanasia, eut**h**anasia) = *1 (adds 1 character h); |*eutanasia| *= 9 and |*euthanasia*| = 10.

We complete the calculation of the Levenshtein distance by the similarity function Stoilos proposed in [20]. It has been specifically developed for strings that are labels of concepts in ontologies. It is based on the idea that the similarity between two entities is related to their commonalities as well as their differences. Thus, the similarity should be a function of both these features. It is defined by the equation (2) where *Comm(S_1_, S_2_) *stands for the commonality between the strings *S_1 _*and *S_2_*, *Diff(S_1_, S_2_) *for the difference between *S_1 _*and *S_2_*, and *Winkler(S_1_, S_2_) *for the improvement of the result using the method introduced by Winkler in [23]:

(2) $$Sim\left(S_{l},S_{2}\right)=Comm\left(S_{l},S_{2}\right)-\mathrm{Diff}\left(S_{l},S_{2}\right)+winkler\left(S_{l},S_{2}\right)$$

The function of commonality is determined by the substring function. The biggest common substring between two strings (*MaxComSubString*) is computed. This process is further extended by removing the common substring and by searching again for the next biggest substring until none can be identified. The function of commonality is given by the equation (3):

(3) $$Comm\left(S_{1},S_{2}\right)=\frac{2\times \underset{i}{\sum }|MaxComSubString_{i}|}{\left|S_{1}|+|S_{2}\right|}$$

For example for the strings *S_1 _*= ***Trigonocep**ah**lie ***and *S_2 _*= ***Trigonocep**ha**lie ***we have: *|MaxComSubString_1_|=|Trigonocep| *= 10; |*MaxComSubString_2_|=|lie| *= 3 *Comm(Trigonocepahlie, Trigonocephalie) *= 0.866.

The difference function *Diff(S_1_, S_2_) *is based on the length of the unmatched strings resulting from the initial matching step. The function of difference is defined in equation (4) where *p *∈ [0, ∞], |*u_S1_| *and |*u_S2_| *represent the length of the unmatched substring from the strings *S_1 _*and *S_2 _*scaled respectively by their length:

(4) $$Di\mathrm{ff}\left(S_{l},S_{2}\right)=\frac{\left|u_{S1}|\times |u_{S2}\right|}{p+\left(1-p\right)\times \left(\left|u_{S1}|+|u_{S2}|-|u_{Sl}|\times |u_{S2}\right|\right)}$$

For example for *S_1 _*= *Trigonocepahlie *and *S_2 _*= *Trigonocephalie *and *p = *0.6 we have: |*u_S1_| = *2/15*; *|*u_S2_| *= 2/15; *Diff(S_1_, S_2_) *= 0.0254.

The Winkler parameter *Winkler(S_1_, S_2_) *is a factor that improves the results [5,23]. It is defined by the equation (5) where *L *is the length of common prefix between the strings *S_1 _*and *S_2 _*at the start of the string up to a maximum of 4 characters and *P *is a constant scaling factor for how much the score is adjusted upwards for having common prefixes. The standard value for this constant in Winkler's work is *P = 0.1 *:

(5) $$Winkler\left(S_{l},S_{2}\right)=L\times P\times \left(1-Comm\left(S_{l},S_{2}\right)\right)$$

For example, *Sim(S_1_, S_2_)*, between the strings *S_1 _= hyperaldoterisme *and *S_2 _= hyperaldosteronisme*. We have *|S_1_| *= 16, *|S_2_| *= 19; the common substrings between *S_1 _*and *S_2 _*are *hyperaldo*, *ter*, and *isme*. *Comm(S_1_, S_2_) *= 0.914; *Diff(S_1_, S_2_) *= 0; *Winkler(S_1_, S_2_) *= 0.034 and *Sim(**hyperaldoterisme***, ***hyperaldoster**on**isme***) = 0.948.

### Processing users' queries

As detailed in [12], spelling errors can be classified as typographic and phonetic. Cognitive errors are caused by a writer's lack of knowledge and phonetic ones are due to similar pronunciation of a misspelled and corrected word. The queries are pre-processed by a phonetic transcription before applying the Levenshtein edit distance along with the similarity function Stoilos.

CISMeF is a quality-controlled health gateway developed at Rouen University Hospital in France [21]. Doc'CISMeF is the search tool associated with CISMeF. Many ways of navigation and information retrieval are possible through the catalogue. The most used is the simple search, with a free text interface. The information retrieval algorithm is based on the subsumption relationships (specialization/generalization) between medical terms, using their hierarchical information, going from the top of the hierarchy to the bottom. If the user query can be matched to an existing term from the terminology, the result is thus the union of the resources indexed by the term, and the resources that are indexed by the terms it subsumes, either directly or indirectly, in all the hierarchies it belongs to. For example, a query on the term *Hepatitis *gives a set of documents indexed by the descriptor *Hepatitis *but also by the descriptors *Hepatitis a, Hepatitis b *and so on. However, the vocabularies of medical terminologies are difficult to apprehend for a user who is not familiar with the domain.

The different materials that we have used to apply the method of spell-checking are related mainly to the search tool Doc'CISMeF: a set of queries and a dictionary of entry terms.

### First set of test queries

We first selected a set of queries sent to Doc'CISMeF by different users. A set of 127,750 queries were extracted from the query log server (3 months logs). Only the most frequent queries were selected. In fact some queries are more frequent than others. For example, the query *"swine flu" *is more present in the query log than *"chlorophyll"*. We eliminated the doubles (68,712 queries remained). From these 68,712 queries, we selected 25,000 queries to extract those with no answers (7,562). From these, we selected queries with misspellings from the most frequent queries in the original set and constituted a first sample test of 163 queries. To avoid phonetic errors of misspelling we first performed a phonetic transcription of this sample with the "*Phonemisation*" function the method of which is detailed below.

### Phonetic transcription of queries and dictionary

Soundex ("Indexing on sound") was the first phonetic string-matching algorithm developed in 1918 [24] for name matching. The idea was to assign common codes to similar sounding names. Intuitively, names referring to the same person have identical or similar Soundex codes. The length of the code is four and it is of the form *letter*, *digit, digit, digit*. The first letter of the code is the same as the first letter of the word. For each subsequent consonant of the word, a digit is concatenated at the end of the code. All vowels and duplicate letters are ignored. The letters *h*, *w *and *y *are also ignored. If the code exceeds the maximum length, extra characters are ignored. If the length of the code is less than 4, zeroes are concatenated at the end. The digits assigned to the different letters for English in the original Soundex algorithm are shown in Table 1: *Soundex*(Robert) = R163; *Soundex*(Robin) = R150 (an extra 0 is added to obtain 3 digits); *Soundex*(Mith) = S530 and Soundex(Smith) = S530.

**Table 1:** Soundex codes

Digits	1	2	3	4	5	6
**Letters**	b, f, p, v	c, g, j, k, q, s, x, z	d, t	L	m, n	r

Many variations of the basic Soundex algorithm, such as changing the code length, assigning a code to the letter of the string or making N-Gram substitutions before code assignment have been tested.

For the French language, Phonex [25] was developed for French names. We present here some variations of the original Phonex algorithm adapted to French medical language, the pronunciation of which is more complex than that of names and bringing together letters according to their type of pronunciation may cause confusion. For example *Phonex*(androstènes*) = Phonex(androstenols) *= 0.082050249 whereas pronunciation is very different (as well meaning). The codes of the *Phonemisation *algorithm are in Table 2.

**Table 2:** Phonemisation codes

Code	Sound	Example
1	"u"[œ]	Comm**un**

2	"oi" [wa]	F**oi**e

3	"ou" [u]	Gen**ou**

4	"en" [ã]	Sci**en**ce

5	"ch"[,ſ]	Bron**ch**e

6	"ill" [j]	Ore**ill**e

7	"gn" [Л]	Soi**gn**er

8	"é" [e] "è" [ε] "e" [ø]	Pr**é**l**è**v**e**ment

0	"oin" [wœ]	S**oin**

The *Phonemisation *function of medical terms that have been developed, allows us to find a word even if it is written with the wrong spelling but with good sound. For example, for the query *"kollesterraulle" *(instead of *"cholesterol"*) *Phonemisation*(kollesterraulle) *= Phonemisation*(cholesterol)*="*kolesterol*"*. We have also constituted manually a list of words that are pronounced *"e" *in French but ending in *"er" *or *"ed"*. To encode the terms, changes are made according to the letters that follow or precede groups of letters that have a particular sound. For example, for the word *"insomnia" *the letters 'in' are replaced by the code '1' giving *Phonemisation*(insomnia) = *"1somnia"*. However, in the word *"inosine" *we also find the same combination of letters '*in*' but, as the next letter "*o*" is a vowel, no changes in the word are made.

We have also considered that in many cases some letters or even combinations of letters are not pronounced at the end of a word. Some combinations are reported in Table 3 modifications in Table 4 and some examples in Table 5. The algorithm of the *Phonemisation *function (detailed in [8]) takes as input a single word and as output another string.

**Table 3:** String modifications according to letters combinations and groups of letters before and after the combination

Combination	Group of Letter		Modification
		
	Before	After	
An		'a','e','i','o','u','n','1','2','3','4','6','8','0'	4

Am		'a','e','i','o','u','n','m','1','2','3','4','6','7','8','0'	4

Ein		'a','e','i','o','u','n','1','2','3','4','6','8','0'	1

Ain		'a','e','i','o','u','n','1','2','3','4','6','8','0'	1

Eim		'a','e','i','o','u','m','1','2','3','4','6','8','0'	1

En		'a','e','i','o','u','n','1','2','3','4','6','8','0'	4

Em		'a','e','i','o','u','m','1','2','3','4','6','8','0'	4

Oin		'a','e','i','o','u','n','1','2','3','4','6','8','0'	0

In	'o', 'e', 'a'	'a','e','i','o','u','n','1','2','3','4','6','8','0'	1

Im	'o', 'e', 'a'	'a','e','i','o','u','m','1','2','3','4','6','8','0'	1

Un		'a','e','i','o','u','n','1','2','3','4','6','8','0'	1

Ge		'a','o','2','3','4','0'	g

Gu		'e','i','1','2','4','6','8','0'	g

**Table 4:** Some modifications according to letters combinations

**Combin**.	**Modif**.	Combin	Modif	Combin	Modif	Combin	Modif	Combin	Modif
sch	5	l1	l8n	irop	iro	qu	k	5t	kt

Ch	5	U	o	irops	iro	s	ss	5l	kl

Sh	5	r0	ro1	thm	m	h	Ø	ptio	psio

Ai	8	omac	oma	stme	sm	31	0	ati4	assi4

Xs	ks	8 mm	am	Am7	ami	ei	8	Oz1	os1

o6	26	si5	sik	tion	sion	oi	2	q	k

oeu	8	gn	7	5o	ko	c	k	5r	kr

**Table 5:** Some sound matching

Word	Phonemisation
Acupuncture	Akup1ktur

Tabac	Taba

Ville	Vil

Sang	S4

In order to compare the sound of two strings, one query and one entry term, all the terms of the dictionary were segmented, lowercased and coded using the function *Phonemisation*. This segmentation is also necessary in cases where for example a user formulates the query *"cretzvelt" *instead of the descriptor *"Creutzfeldt-Jakob"*. The function *Phonemisation *was performed on the set of 163 queries as a preliminary stage before spell-checking by combining the Levenshtein edit distance and the Stoilos similarity function. The reference dictionary (the structure of which is detailed in Table 6) was created between 1995 and 2005 exclusively on the French version of the MeSH thesaurus [26] maintained by the US National Library of Medicine, completed by numerous synonyms in French collected by the CISMeF team.

**Table 6:** Composition of the reference dictionary based on the MeSH in French

	MeSH Terms	MeSH Synonyms	CISMeF synonyms	Total
**1 word**	9,679	9,391	3,359	22,429

**2 words**	9,833	28,051	8,258	46,142

**3 words**	4,204	19,551	6,569	30,324

**4 words and +**	2,503	16,992	4,924	24,419

### Second sample of test queries: multi-word queries

The second set of test queries was constituted to evaluate spell-checking on a larger scale. A set of 6,297 frequent queries was constituted from the original set of 7,562. In this set, the queries were composed from 1 to 4 and more words (see Table 7). To process multi-word queries, we used basic natural language processing steps and the well-known Bag-of-Words (BoW) algorithm:

**Table 7:** Structure of the queries (with no answer) obtained from the logs

Composition	Number
1 word	1,061

2 words	1,636

3 words	1,443

4 (and more) words	2,157

**Total**	**6,297**

#### Query segmentation

The query was segmented in words thanks to a list of segmentation characters and *string tokenizers*. This list is composed of all the non-alphanumerical characters (*e.g*.: * $,!§;|@).

#### Character normalizations

We applied two types of character normalization at this stage. MeSH terms are in the form of non-accented uppercase characters. Nevertheless, the terms used in the CISMeF terminology are in mixed-case and accented. (1) *Lowercase conversion*: all the uppercased characters were replaced by their lowercase version; "*A*" was replaced by "*a*". This step was necessary because the controlled vocabulary is in lowercase. (2) *Deaccenting*: all accented characters (*"éèêë"*) were replaced by non-accented (*"e"*) ones. Words in the French MeSH were not accented, and words in queries were either accented or not, or wrongly accented (*h**è**patite" *instead *"h**é**patite"*).

#### Stop words

We eliminated all stop words (such as *the, and, when*) in the query. Our stop word list was composed 1,422 elements in French (*vs*. 135 in PubMed).

#### Exact expression

We use regular expressions to match the exact expression of each word of the query with the terminology. This step allowed us to take into account the complex terms (composed of more than one word) of the vocabulary and also to avoid some inherent noise generated by the truncations. The query *'**accident**' *is matched with the term *'circulation ****accident***' but not with the terms *'**accident**' *and '*chute **accident**elle*'. The query '***sida***' is matched with the terms *'lymphome lié **sida***' and *'**sida **atteinte neurologique' *but not with the terms *'gluco**sida**ses'*, *'agra**sida**e' *and *'bêta galacto**sida**se'*.

#### Phonemisation

The function is as described in the previous section. It converts a word into its French phonemic transcription: *e.g*. the query *alzaymer *is replaced by the reserved term *alzheimer*.

#### Bag of words

The algorithm searched the greatest set of words in the query corresponding to a reserved term. The query was segmented. The stop words were eliminated. The other words were transformed with the *Phonemisation *function and sorted alphabetically. The different reserved term bags were formed iteratively until there were no possible combinations. The query *'therapy of the breast cancer' *gave two reserved words: '*therapeutics' *and *breast cancer' *(*therapy *being a synonym of the reserved term *therapeutics)*.

### Evaluations

To evaluate our method of correcting misspellings, we used the standard measures of evaluation of information retrieval systems, by calculating precision, recall and the F-Measure. We performed a manual evaluation to determine these measures. Precision (6) measured the proportion of queries that were properly corrected among those corrected.

(6) $$Precision=\frac{\left|\left\{Queries\;correctly\;corrected\right\}\right|}{\left|\left\{Queries\;corrected\right\}\right|}$$

Recall (7) measured the proportion of queries that were properly corrected those requiring correction.

(7) $$Recall=\frac{\left|\left\{Queries\;correctly\;corrected\right\}\right|}{\left|\left\{Queries\;to\;be\;corrected\right\}\right|}$$

The F-Measure combined the precision and recall by the following equation:

(8) $$F-Measure=\frac{2\times Precision\times Recall}{\left(Precision+Recall\right)}$$

We also calculated confidence intervals at ρ = 5% to avoid evaluating the whole set of queries, but some sets that are manually manageable. For a proportion *x *and a set of size *n_x _*the confidence interval is:

(9) $$CI_{x}=\left[x-1.96\times \sqrt{\frac{x\times \left(1-x\right)}{n_{x}}};x+1.96\times \sqrt{\frac{x\times \left(1-x\right)}{n_{x}}}\right]$$

## Results

### Choice of thresholds for the first set of queries

**Figure 1:**
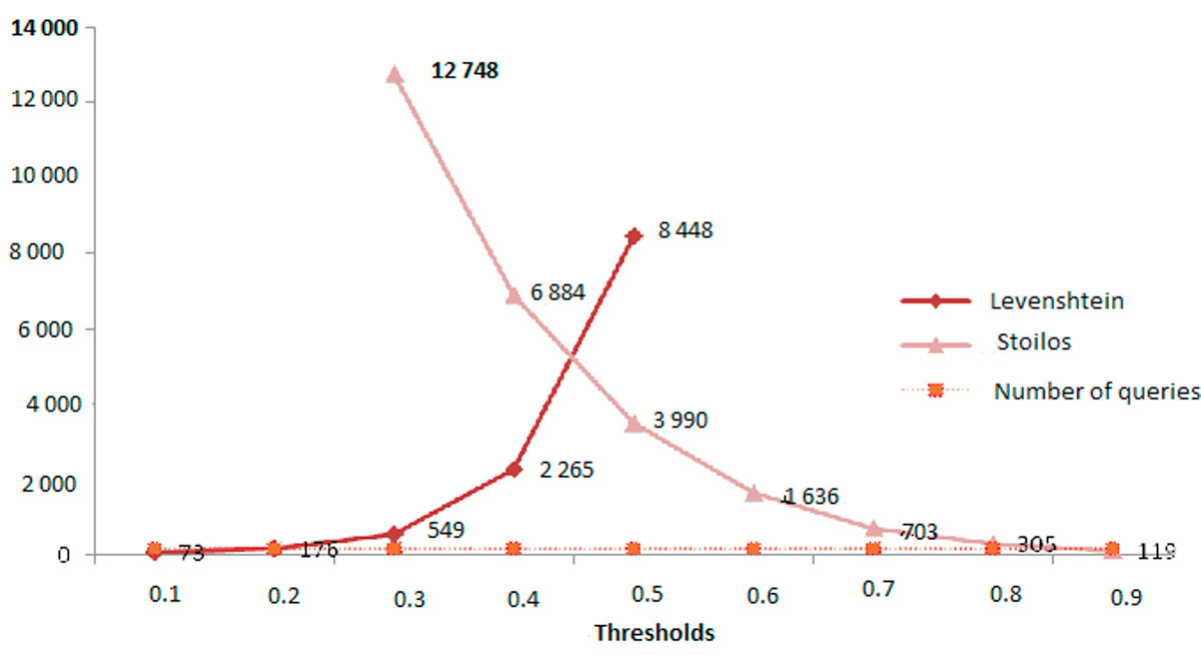
**Total number of suggestions according to different thresholds of Levenshtein and Stoilos**.

The Levenshtein and Stoilos functions require a choice of thresholds to obtain a manageable number of correction suggestions for the user. We thus tested different thresholds, as shown in Tables 8, 9 and 10 and Figure 1, for the normalized Levenshtein distance, the similarity function of Stoilos and for the combination of both. For example, the query "*ac**c**up**o**nture*" (instead *a**c**up**u**n**c**ture*) is corrected with Levenshtein < 0.3. At a threshold of 0.6, 120 suggestions are proposed. The same query is corrected with Stoilos > 0.5 and at a threshold of 0.1, 56 suggestions are proposed. When combining Lev < 0.3 and Stoilos > 0.1 only one (and correct) suggestion is proposed. The query "*suette*" (instead *suette miliaire *(sweating sickness)) is corrected properly with Levenshtein < 0.6 (224 suggestions for this query), Stoilos > 0.7 (2 suggestions) and with Levenshtein < 0.8 combined with Stoilos > 0.1 (114 sugestions). The query *"rickttsiose*" (instead *rickettsioses *(Rickettsia infections) is corrected properly with Levenshtein < 0.15 (1 suggestion), Stoilos > 0.9 (1 suggestion) and with Levenshtein < 0.2 combined with Stoilos > 0.9 (1 suggestion).

**Table 8:** Numbers of proposed corrections with the Levenshtein edit distance at different thresholds

Thresholds	< 0.05	< 0.1	< 0.15	< 0.2	< 0.25	< 0.3	< 0.35	< 0.4	< 0.45	< 0.5	< 0.6	< 0.7	< 0.8	< 0.9
**Suggestions**	14	73	118	176	273	549	1,187	2,265	4,707	8,448	59,844	656,291	5,368,088	13,695,608

**Nb by query**	0.08	0.44	0.72	1.07	1.67	3.36	7.28	13.89	28.87	51.83	367.14	4,026.32	32,933	84,022

**Table 9:** Numbers of proposed corrections with the Stoilos function at different thresholds

Thresholds	> 0.1	> 0.2	> 0.3	> 0.4	> 0.5	> 0.6	> 0.7	> 0.8	> 0.9
**Suggestions**	42,721	23,658	12,748	6,884	3,490	1,636	703	305	119

**Nb by query**	262.09	145.14	78.2	42.23	21.41	10.03	4.31	1.87	0.73

**Table 10:** Numbers of proposed corrections (between brackets the number by query) at different thresholds with the Stoilos function combined with the Levenshtein edit distance

		Levenshtein										
		
		< 0.05	< 0.1	< 0.15	< 0.2	< 0.3	< 0.4	< 0.5	< 0.6	< 0.7	< 08	< 0.9
**Stoilos**	**> 0.1**	6 (0.03)	63 (0.38)	107 (0.65)	165 (1.01)	538 (3.30)	2,188 (13.42)	6,563 (40.20)	18,274 (112.11)	30,303 (185.90)	39,456 (242.06)	42,483 (260.63)
	
	**> 0.2**	6 (0.03)	63 (0.38)	107 (0.65)	165 (1.01)	537 (3.29)	2,118 (12.99)	5,806 (35.61)	13,053 (80.79)	18,790 (115.27)	22,395 (137.39)	23,576 (144.63)
	
	**> 0.3**	6 (0.03)	63 (0.38)	107 (0.65)	165 (1.01)	534 (3.27)	1,990 (12.20)	4,680 (28.71)	8,352 (51.23)	10,909 (66.92)	12,328 (75.63)	12,709 (77.96)
	
	**> 0.4**	6 (0.03)	63 (0.38)	107 (0.65)	165 (1.01)	526 (3.22)	1,789 (10.97)	3,548 (21.76)	5,262 (32.28)	6,236 (38.25)	6,749 (41.40)	6,864 (42.11)
	
	**> 0.5**	6 (0.03)	63 (0.38)	107 (0.65)	164 (1.00)	492 (4.92)	1,397 (8.57)	2,313 (14.19)	2,910 (17.85)	3,268 (20.04)	3,435 (21.07)	3,478 (21.33)
	
	**> 0.6**	6 (0.03	63 (0.38)	107 (0.65)	162 (0.99)	431 (2.64)	864 (5.30)	1,199 (7.35)	1,431 (8.77)	1,562 (9.58)	1,617 (9.92)	1,625 (9.96)
	
	**> 0.7**	6 (0.03)	63 (0.38)	106 (0.65)	160 (0.98)	292 (1.79)	448 (2.74)	556 (3.41)	653 (4.0)	685 (4.20)	690 (4.23)	692 (4.24)
	
	**> 0.8**	6 (0.03)	62 (0.38)	97 (0.59)	138 (0.84)	182 (1.11)	231 (1.41)	275 (1.68)	288 (1.76)	290 (1.77)	293 (1.79)	294 (1.80)
	
	**> 0.9**	6 (0.03)	52 (0.31)	79 (0.48)	95 (0.58)	103 (0.63)	105 (0.64)	106 (0.65)	106 (0.65)	106 (0.65)	108 (0.66)	108 (0.66)

As shown in Tables 8, 9 and 10 and Figure 1, the number of suggestions provided to the user in order to correct is variable and the task of correcting queries may become overwhelming if the user has to select the correct word from hundreds, even millions (for Levenshtein < 0.9). Manageable results (around 163, the number of queries) are obtained for the following thresholds for (i) Levenshtein < 0.3; (ii) Stoilos > 0.7 and (iii) the combination of Lenshtein < 0.3 and Stoilos > 0.6.

### Evaluation on the first sample of queries

We first tested the method with standard Levenshtein with thresholds from 0.05 to 0.6. Manual evaluation gave from 14 queries corrected without any error, to 163 queries corrected, 22 with false suggestions. Precision decreased from 100 to 86.50% and recall increased from 08.58% to 86.50%. The best F-Measure is obtained for Levenshtein < 0.4 (88.95%). However, for this threshold, the total number of suggestions is 2,265 (Table 11). We tested the method with Stoilos function with thresholds from 0.1 to 0.9.

**Table 11:** Evaluations and numbers of corrected queries for Levenshtein edit distance with different thresholds

Threshold	< 0.05	< 0.1	< 0.15	< 0.2	< 0.25	< 0.3	< 0.35	< 0.4	< 0.45	< 0.5	< 0.6
**Number of suggestions**	14	73	118	176	273	549	1,187	2,265	4,707	8,448	59,844

**Answered Queries**	14	71	105	126	137	141	148	154	157	162	163

**Precision (%)**	100	100	99.04	97.61	95.62	95.03	91.89	91.55	89.80	87.03	86.50

**Recall (%)**	08.58	43.55	63.80	75.46	80.36	82.20	83.43	86.50	86.50	86.50	86.50

**F-Measure (%)**	15.81	60.68	77.61	85.12	87.33	88.15	87.45	88.95	88.12	86.76	86.50

**Figure 2:**
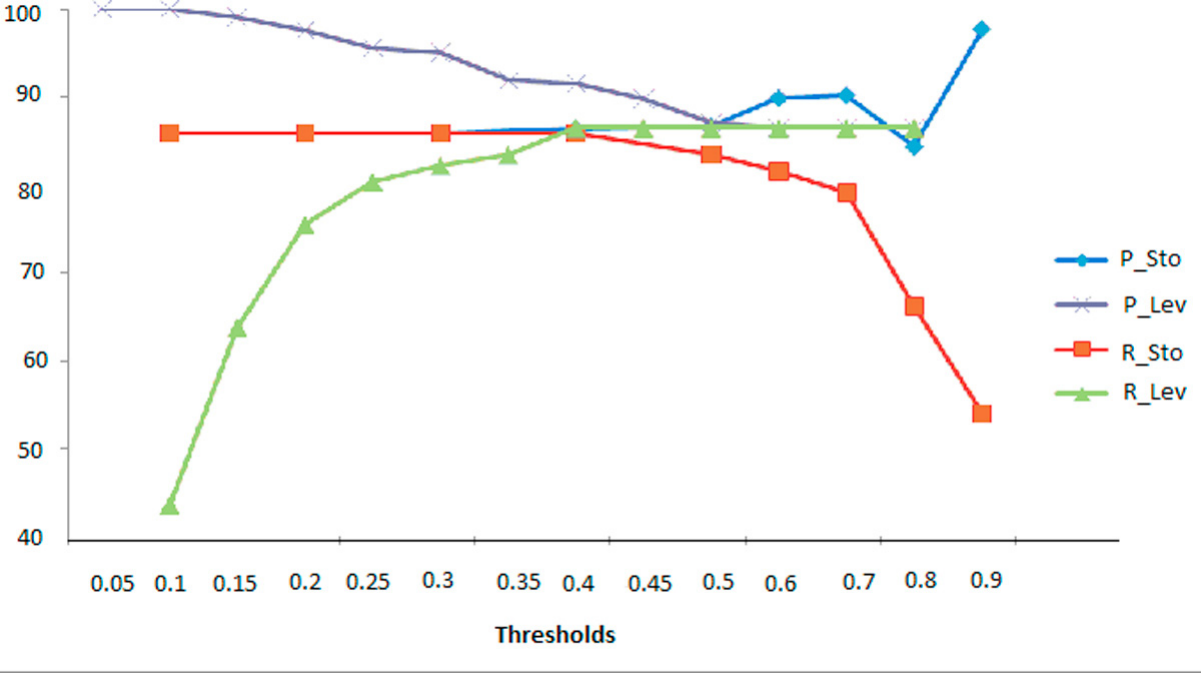
**Precision (P) and recall (R) curves according to different thresholds of Levenshtein (Lev) and Stoilos (Sto)**.

Manual evaluation gave from 163 queries corrected, 23 with false suggestions, to 90 queries corrected, 2 with false suggestions. Precision increased from 85.88% to 97.77% and recall decreased from 85.88% to 53.98. The best F-Measure is obtained for Stoilos > 0.4. However, for this threshold the total number of suggestions is 6,884 (Table 12). The resulting curves of precision and recall of Stoilos and Levenshtein according to different thresholds are in Figure 2.

**Table 12:** Evaluations and numbers of corrected queries for Stoilos function with different thresholds

Threshold	> 0.9	> 0.8	> 0.7	> 0.6	> 0.5	> 0.4	> 0.3	> 0.2	> 0.1
**Number of suggestions**	119	305	705	1,636	3,490	6,884	12,748	23,659	42,721

**Answered Queries**	90	128	143	148	157	162	163	163	163

**Precision (%)**	97.77	84.37	90.20	89.86	86.62	86.41	85.88	85.88	85.88

**Recall (%)**	53.98	66.25	79.14	81.59	83.43	85.88	85.88	85.88	85.88

**F-Measure (%)**	69.56	74.22	84.31	85.55	85.00	86.15	85.88	85.88	85.88

**Figure 3:**
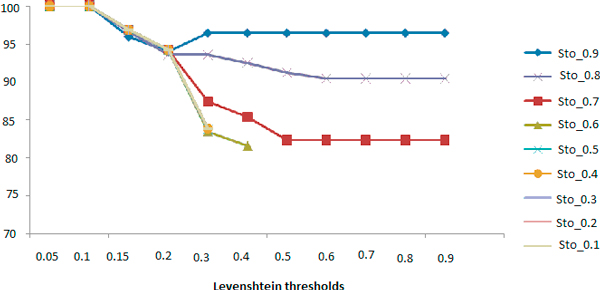
**Precision curves according to different thresholds of Levenshtein combined with Stoilos (Sto) with different thresholds**.

**Figure 4:**
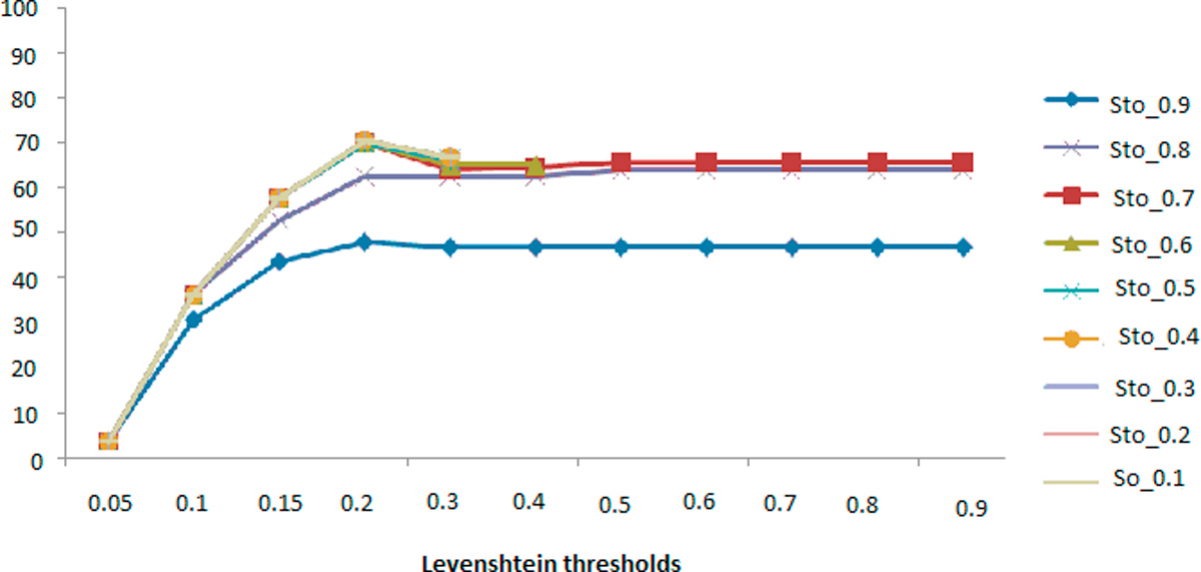
**Recall curves: Levenshtein combined with Stoilos**.

We also tested the combination of Stoilos along Levenshtein. Manual evaluations were not performed on all the possible combinations (Table 13). Figure 3 and 4 contain resulting curves of precision and recall respectively.

**Table 13:** Evaluation (P: Precision, R: Recall, F: F-Measure) and number of corrected queries (Q) with Levenshtein and Stoilos combinations

		Levenshtein										
		
		< 0.05	< 0.1	< 0.15	< 0.2	< 0.3	< 0.4	< 0.5	< 0.6	< 0.7	< 0.8	< 0.9
**Stoilos**	**> 0.9**		Q:50 P = 100 R = 30.67 F = 46.94	Q:74 P = 95.94 R = 43.55 F = 59.91	Q:83 P = 93.97 R = 47.85 F = 63.41				Q:84 P = 96.42 R = 46.69 F = 65.58			
			
	**> 0.8**			Q:89 P = 96.62 R = 52.76 F = 68.25	Q:109 P = 93.57 R = 62.57 F = 75.00		Q:110 P = 92.72 R = 62.57 F = 74.72	Q:114 P = 91.20 R = 63.81 F = 75.09		Q:115 P = 90.43 R = 63.80 F = 74.82		
				
	**> 0.7**	Q: 6 P = 100 R = 03.60 F = 07.10				Q:119 P = 87.39 R = 63.80 F = 73.75	Q:123 P = 85.36 R = 64.41 F = 73.42	Q:130 P = 82.30 R = 65.64 F = 73.03				
						
	**> 0.6**		Q:59 P = 100 R = 36.19 F = 53.15	Q:97 P = 96.90 R = 57.66 F = 72.30	Q:121 P = 94.21 R = 69.93 F = 80.28	Q:127 P = 83.46 R = 65.03 F = 73.10	Q:130 P = 81.53 R = 65.03 F = 72.35					
											
	**> 0.5**					Q:129 P = 83.72 R = 66.25 F = 73.97		Not evaluated				
											
	**> 0.4**				Q:122	Q:130						
												
	**> 0.3**				P = 94.26	P = 83.84						
												
	**> 0.2**				R = 70.55	R = 66.87						
												
	**> 0.1**				F = 80.70	F = 74.75						

Note that the function *Phonemisation *gave a 38% recall, 42% precision and 39.90% F-Measure, which are lower than the methods based on string edit distance or similarity function.

According to all those results (mainly precision, total number of suggestions and number of corrected queries) we retained a threshold of 0.2 for Levenshtein edit distance and 0.7 for Stoilos function, when combinated for spelling-correction.

**Figure 5:**
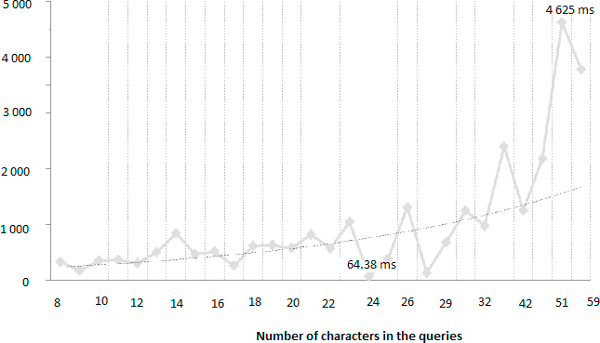
**Times according to the size of the queries with Lev < 0.2 and Sto > 0.7**.

We also measured the time necessary to propose spelling-corrections to information seekers according to the size of the queries, using Levenshtein < 0.2 along with Stoilos > 0.7 and we obtained at min: 64.38 ms and at max : 4,625 ms (Figure 5).

### Evaluation of the second sample of queries

**Figure 6:**
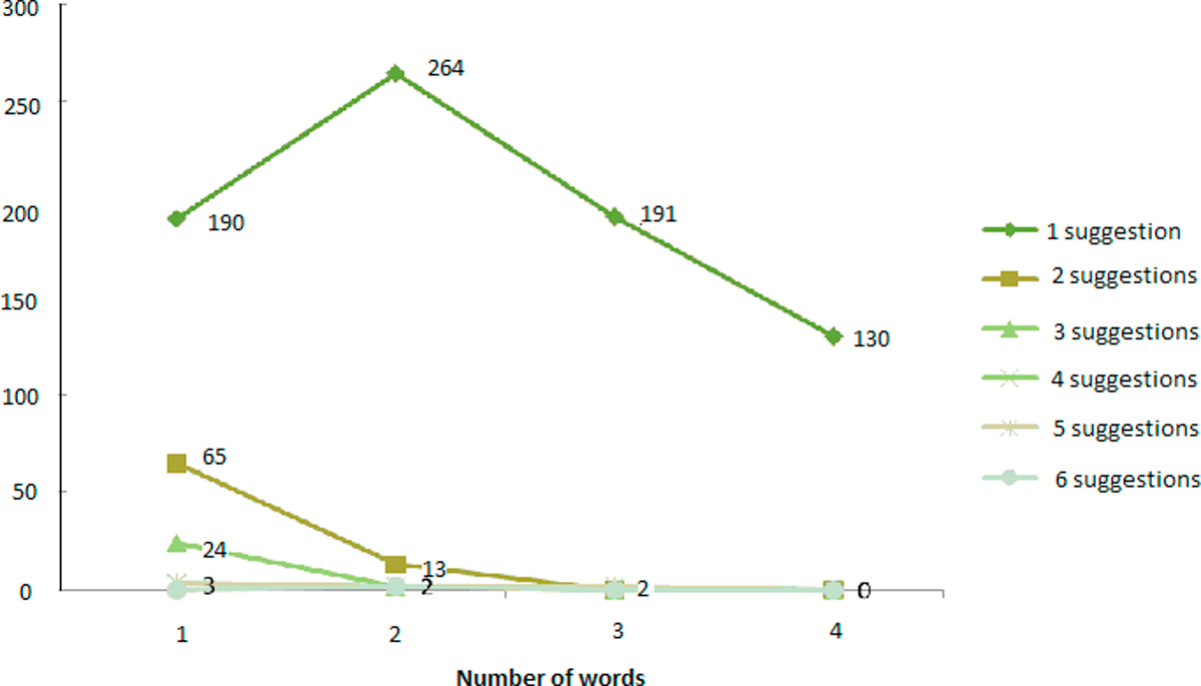
**Total number of suggestions according to the size of the query**.

The second set of queries was larger (6,297) and composed of queries of 1 to 4 and more words. In this evaluation, we chose to retain the following thresholds: Levenshtein > 0.2 and Stoilos > 0.7. To determine the impact of the size of the query we measured the number of suggestions of corrected queries (Figure 6 and Table 14). For a user, the maximum number of manageable suggestions for one query was 6.

**Table 14:** Number of suggestions according to the size of the query

	Nb characters	Nb suggestions by query
**1 word query**	Min = 3; Avg = 10.49; Max = 25	Avg = 0.39; Max = 5

**2 words query**	Min = 5; Avg = 18.36; Max = 41	Avg = 0.22; Max = 6

**3 words query**	Min = 10; Avg = 24.39; Max = 54	Avg = 0.13; Max = 1

**4 words and +query**	Min = 11; Avg = 37.30; Max = 113	Avg = 0.06; Max = 1

Manual evaluations were performed on sets of ~1/3 of each type of queries. Table 15 contains all the Precison, Recall and F-Measure values. Evaluations of the quality of queries suggestions (Precision, Recall and F-Measure) were performed manually on several sets, according to the size of the query, but also according to the following methods : Bag-of-Words, Levenshtein distance alongside the Stoilos similarity function, but also the Bag-of-Words processed before and after the combination of the Levenshtein distance along with the Stoilos similarity function. Levenshtein and Stoilos remained constant at < 0.2 and > 0.7 respectively.

**Table 15:** Evaluation measures of the different methods : Bag-of-Words (BoW), Levenshtein along with Stoilos (LS), LS performed before BoW, and BoW performed before Levenshtein combined with Stoilos

	1 word set of 310 queries among 1,061)			2 words (set of 450 queries among 1,636)			3 words (set of 594 queries among 1,443)			4 words + (set of 710 queries among 2,157)			Total (set of 2,064 queries among 6,297)		
	
	P(%)	R(%)	F(%)	P(%)	R(%)	F(%)	P(%)	R(%)	F(%)	P(%)	R(%)	F(%)	P(%)	R(%)	F(%)
**BoW**	**100**	26.85	42.33	**100**	34.81	51.64	**100**	44.06	61.17	**100**	38.16	55.24	**100**	35.88	52.81
	
	[100-100]	[19.73-33.96]	[32.96-50.70]	[100-100]	[27.38-42.24]	[42.99-59.39]	[100-100]	[35.92-52.19]	[52.85-68.59]	[100-100]	[30.44-45.88]	[46.67-62.90]	[100-100]	[32.05-39.71	[48.54-56.85]

**LS**	92.11	46.98	62.22	82.61	36.08	50.22	51.56	23.08	31.88	46.77	11.18	18.05	69.74	29.40	41.37
	
	[86.04-98.17]	[38.97-54.99]	[53.64-70.49]	[73.67-91.55]	[28.59-43.56]	[40.76-59.03]	[39.32-63.81]	[16.17-29.98]	[22.92-40.79]	[34.35-59.19]	[6.17-16.19]	[10.46-25.43]	[64.27-75.21]	[25.76-33.04]	[36.78-45.91]

**LS before BoW**	93.10	54.36	68.64	83.78	39.24	53.45	58.67	27.97	37.88	51.47	12.50	20.1	73.03	30.40	42.93
	
	[87.78-98.43	[46.36-62.36]	[60.68-76.35]	[75.39-92.18]	[31.63-46.85]	[44.56-62.13]	[47.52-69.81]	[20.62-35.33]	[28.76-46.92]	[39.59-63.35]	[7.24-17.76]	[12.24-27.74]	[68.04-78.02]	[26.72-34.07]	[38.37-47.43]

**BoW before LS**	86.67	**61.07**	**71.65**	84.96	**60.76**	**70.85**	65.65	**60.14**	**62.77**	72.92	**46.05**	**56.45**	77.08	**54.98**	**64.18**
	
	[80.16-93.17]	[53.24-68.9]	[63.98-79.22]	[78.36-91.55	[53.15-68.37]	[63.34-78.28]	[57.52-73.78]	[52.11-68.16]	[54.68-70.86]	[64.03-81.81]	[38.13-53.98]	[47.80-65.04]	[73.17-80.98]	[51.01-58.96]	[60.11-68.24]

**Figure 7:**
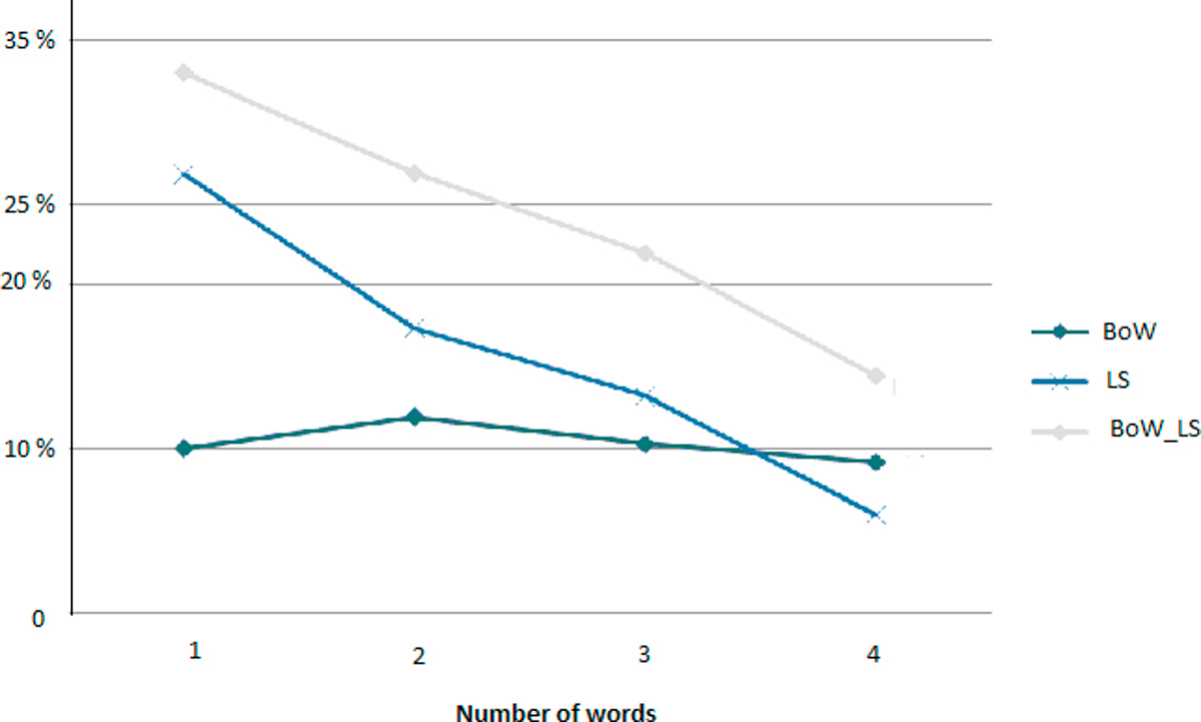
**Proportion of matched queries according to the method and the size of the query : Bag-of-Words (BoW), Levenshtein alongside Stoilos (LS) and BoW with LS**.

By combining the Bag-of-Words algorithm along with the Levenshtein distance and the similarity function of Stoilos, a total of 1,418 (22.52%) queries matched medical terms or combinations of medical terms. The remaining queries with no suggestions (when terms and also the possible combination of terms) not belong to the dictionary. For 1-word queries, it remained 711 (67%), for 2-words queries it remained 1197 queries (73.16%); for 3-words queries it remained 1126 (78.08%) and for 4 words queries it remained 1,846 queries (85.58%) (see Figure 7). For example, the query "*nutrithérapie*" (nutritherapy) contains no error but cannot be matched with any medical term in the MeSH thesaurus.

**Figure 8:**
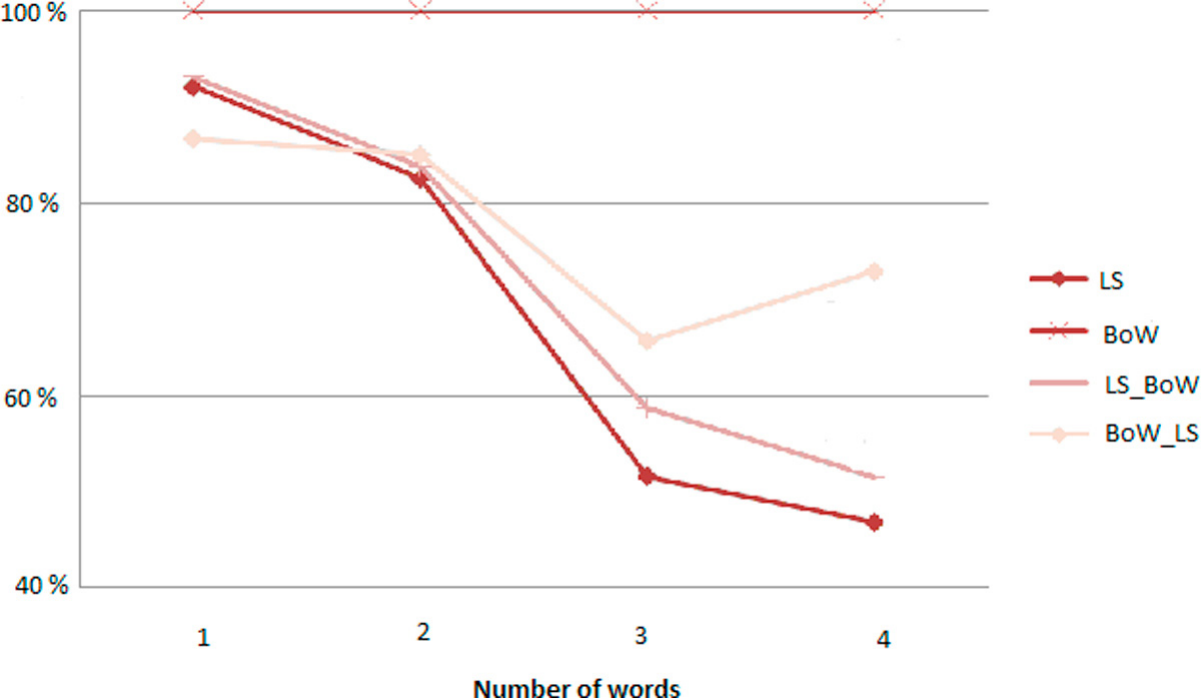
**Precision curves according to the size of the query**.

**Figure 9:**
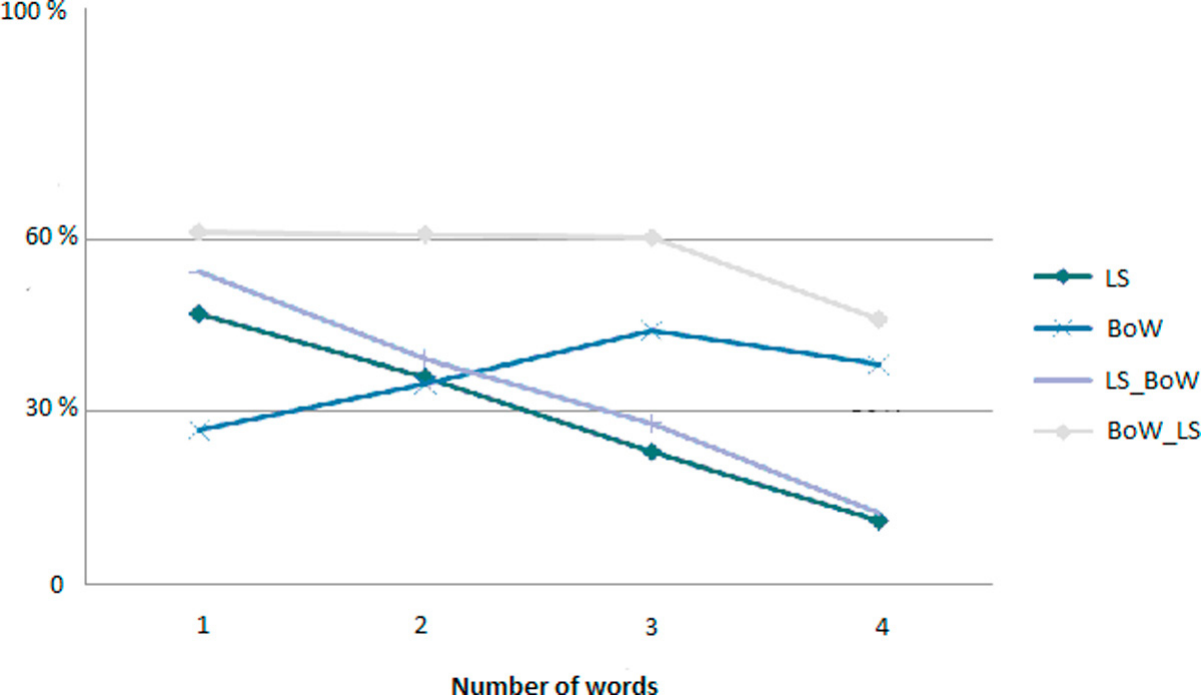
**Recall curves according to the size of the query**.

**Figure 10:**
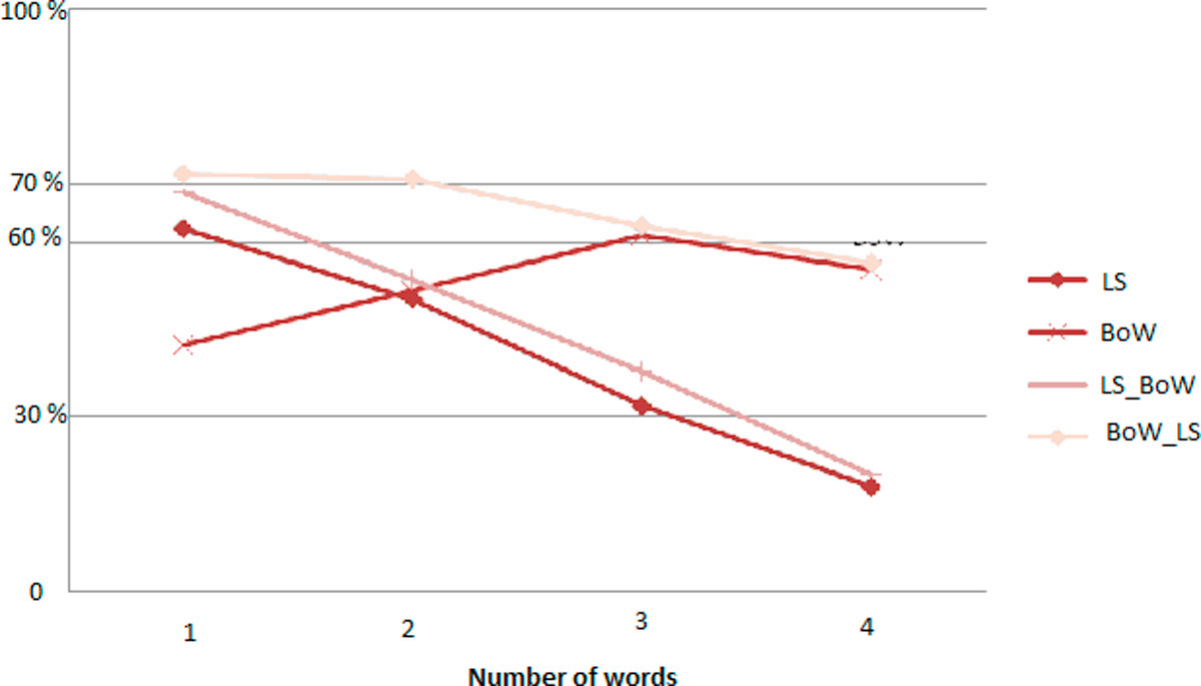
**F-Measure curves according to the size of the query**.

Evaluations shown that best results were obtained by performing the Bag-of-Words algorithm before the combination of Levenshtein alongside Stoilos. The resulting curves of precision, recall anf F-measure are in Figures 8, 9 and 10 respectively.

## Discussion

Several studies have explored the problem of spelling corrections, but the literature is quite sparse in the medical domain, which is a distinct problem, because of the complexity of medical vocabularies. Nonetheless, the work of [27] uses word frequency based sorting to improve the ranking of suggestions generated by programs such as GNU Gspell and GNU Aspell. This method does not detect any misspellings nor generate suggestions but reports that Aspell gives better results than Gspell. In [28] Ruch studied contextual spelling correction to improve the effectiveness of a health Information Retrieval system. In [29] the authors created a prototype spell checker using UMLS and WordNet in English sources of knowledge for cleaning reports on adverse events following immunization. We also cite the work of [30] which proposes a program for automatic spelling correction in mammography reports. It is based on edit distances and bi-gram probabilities but is applied to a very specific sub-domain of medicine, and not to queries but to plain text. In [18] the authors use normalization techniques, aggressive reformatting and abbreviation expansion for unrecognized words as well as spelling correction to find the closest drug names within RxNorm for drug name variants found in local drug formularies. The spelling algorithm is that of the RxNorm API which returns only drug name suggestions. The unknown word must have a minimum length of five characters for spelling correction to be tried. However, the effective usage of the spelling correction component was only 7.6% in the approximate matching of drug names. In addition many spelling corrections were applied to unknown tokens which were not intended to be drugs. The different experiments we performed show that with 38% recall and 42% precision, *Phonemisation *cannot correct all errors : it can only be applied when the query and entry term of the vocabulary have similar pronunciation. However, when there is reversal of characters in the query, it is an error of another type : the sound is not the same and similarity distances such as Levenshtein and Stoilos can be exploited here. Similarly, when using certain characters instead of others ("*ammidale*" instead of "*amygdale*"), string similarity functions are not efficient. The best results (F-Measure 64.18%) are obtained with multi-word queries by performing the Bag-of-Words algorithm first and then the spelling-correction based on similarity measures. Due to the relatively small number of correction suggestions (min 1 and max 6), which are manually manageable by a health information seeker, we have chosen to return an alphabetically sorted list rather than ranking them.

## Conclusions

The general idea of spelling correction is based on comparing the query with either dictionaries or controlled vocabularies. If a query does not match the vocabulary, one or more suggestions are proposed to the user. Recent research has focused on the development of algorithms in recognizing a misspelled word, even when the word is in the dictionary, and based on the calculation of similarity distances. Damerau [10] indicated that 80% of all spelling errors are the result of (i) transposition of two adjacent letters (*ashtma *vs. *asthma*) (ii) insertion of one letter (*asthmma *vs. *asthma*) (iii) deletion of one letter (*astma *vs. *asthma*) and (iv) replacement of one letter by another (*asthla *vs. *asthma*). Each of these wrong operations costs 1 *i.e*. the distance between the misspelled and the correct word.

In this paper, we present a method to automatically correct misspelled queries submitted to a health search tool that may be used both by patients but also by health professionals such as physicians during their clinical practice. We have described how to adapt the Levenshtein and Stoilos to calculate similarity in spell-checking medical terms when there is character reversal. We have also presented the combined approach of two similarity functions and defined the best thresholds. Our results show that using these distances improves phonetic transcription results. This latter step is not only necessary but is less expensive than calculating distance. The best results (in terms of quality and quantity) are obtained by performing the Bag-of-Words algorithm (which includes phonetic transcription) before the combination of Levenshtein and Stoilos similarity functions.

The use of keyword configuration, by studying the distances between keys, is another possible direction to suggest spelling corrections. For example, when the user types a "Q" instead of an "A" which is located just above on the keyboard, similarly to the work detailed in [31] for correcting German brand names of drugs. These errors are more frequent when queries are submitted by a Tablet PC or a smart phone, the keyboard being smaller in size.

This method may also be used to extract medical information from clinical free texts of electronic health records or discharge summaries. Indeed, the efforts to recognize medical terms in text have focused on finding disease names in electronic medical records, discharge summaries, clinical guideline descriptions and clinical trial summaries. The survey of Meystre *et al. *[32] describes several studies on detecting information elements in clinical texts using natural language processing and show their impact on clinical practice. These information elements may be diseases [33], treatments [34] in English, or other medical information in French [35]. However, as in any free text, clinical notes may contain misspellings. Using our method may be a preliminary step to cleaning these notes before coding. The algorithms we have presented in this paper will be integrated into the first work package of the following two research projects, both of which are funded by the French National Research Agency: the RAVEL project for information retrieval through patient medical records and the SIFADO project for helping health professionals to code discharge summaries, which free-text components require manual processing by human encoders.

## Competing interests

The authors declare that they have no competing interests.

## Authors' contributions

LFS, EPG, TL and SJD formulated the idea of this study. LFS, EPG, TL and SJD designed it and participated in writing the draft. ZM designed the first part of the method (queries of one word) and ZM and SJD evaluated it. LFS designed the second part of the method and evaluated with SJD. All authors read and approved the final manuscript.

## References

[B1] Keselman A Browne AC Kaufman DR Consumer health information seeking as hypothesis testing Journal of American Medical Informatics Association 2008 51 4 484 495 10.1197/jamia.M2449 PMC2442260 18436912

[B2] Koch T Quality-controlled subject gateways: definitions, typologies, empirical overview Online Information Review 2000 24 1 24 34 10.1108/14684520010320040

[B3] Abad Garcia F A comparative study of six European databases of medically-oriented web resources Journal of the Medical Library Association 2005 93 4 467 479 16239943 PMC1250323

[B4] McCray AT Ide NC Loane RR Tse T Strategies for supporting consumer health information seeking Proceedings of the 11th World Congress on Health (Medical) Informatics, Medinfo: 7-11 September 2004 2004 San Francisco 1152 56 15360993

[B5] Grannis SJ Overhag MJ Mc Donald C Real world performance of approximate string comparators for use in patient matching Studies in Health Technolgy and Informatics 2004 107 43 47 15360771

[B6] Levenshtein VI Binary codes capable of correcting deletions, insertions and reversals Soviet Physics Dokl 1966 10 707 710

[B7] Yarkoni T Balota D Yap M Moving beyond Coltheart's N: a new measure of orthographic similarity Psychonomic Bulletin & Review 2008 15 5 971 979 10.3758/PBR.15.5.971 18926991

[B8] Soualmia LF Etude et évaluation d'approches multiples d'expansion de requêtes pour une recherche d'information intelligente: application au domaine de la santé sur l'Internet PhD thesis 2004 INSA Rouen

[B9] Hodge VJ Austin J A comparison of a novel neural spell checker and standard spell checking algorithms Pattern Recognition 2002 11 35 2571 2580

[B10] Damereau FJ A technique for computer detection and correction of spelling errors Communication of the ACM, March 1964 7 3 New York 171 177

[B11] Peterson LJ A note on undetected typing errors Communications of ACM 1986 29 7 633 637 10.1145/6138.6146

[B12] Kuckich K Techniques for automatically correcting words in text ACM Comput Surv 1992 24 4 377 439 10.1145/146370.146380

[B13] Kernigham M A spelling correction program based on noisy channel modelproceedings of COLING 90 the 13th International Conference on computational linguistics 1990 2

[B14] Brill E Moore RC An improved error model for noisy channel spelling correctionproceedings of 38th Annual meeting of association for computational linguistics 2000 286 293

[B15] Toutanova K Moore RC Pronunciation Modeling for Improved Spelling Correction Proceedings of the 40th Meeting of the Association for Computational Linguistics (ACL-2002) 141 151

[B16] Boyer C Baujard V Griesser V Scherrer JR HONselect: a multilingual and intelligent search tool integrating heterogeneous web resources International Journal of Medical Informatics 2001 64 2-3 253 258 10.1016/S1386-5056(01)00194-0 11734390

[B17] Crowell J Long Ngo QNG Lacroix E A frequency-based technique to improve the spelling suggestion rank in medical queries Journal of the American Medical Informatics Association 2004 11 3 179 185 10.1197/jamia.M1474 14764616 PMC400516

[B18] Peters L Kapunsik-Uner JE Nguyen T Bodenreider O An approximate matching method for clinical drug name AMIA Annual Symposium 2011 in press PMC3243188 22195172

[B19] Wilbur JW Kim W Xie N Spelling correction in the PubMed search engine Information retrieval 2006 9 543 564 10.1007/s10791-006-9002-8 18080004 PMC2137159

[B20] Stoilos G Stamou G Kollias S A string metric for ontology alignment Proceedings of the International Semantic Web Conference, 6-10 November 2005 2005 Galway 624 637

[B21] Douyère M Soualmia LF Névéol A Enhancing the MeSH thesaurus to retrieve French online health resources in a quality-controlled gateway Health Information Library Journal 2004 4 21 253 61 10.1111/j.1471-1842.2004.00526.x 15606883

[B22] Yujian L Bo L A normalized Levenshtein distance metric IEEE Transactions on Pattern Analysis and Machine Intelligence 2007 29 6 1091 1095 17431306 10.1109/TPAMI.2007.1078

[B23] Winkler W The state record linkage and current research problems Technical report: Statistics of Income Division, Internal Revenue Service Publication 1999

[B24] Stanier A How accurate is Soundex matching Computers in Genealogy 1990 3 7 286 288

[B25] Brouard F L'art des Soundex 2004 http://sqlpro.developpez.com/cours/soundex/

[B26] Nelson SJ Relationships in Medical Subject Heading Relationships in the Organization of Knowledge 2001 171 184

[B27] Gaudinat A Ruch P Joubert M Uziel P Strauss A Thonnet M Health search engine with e-document analysis for reliable search results International Journal of Medical Informatics 2006 75 1 73 85 10.1016/j.ijmedinf.2005.11.002 16377235

[B28] Ruch P Using contextual spelling correction to improve retrieval effectiveness in degraded text collections Proceedings of the 19th International conference on Computational Linguistics 2002 1 7

[B29] Tolentino HD Matters MD Walop W A UMLS-based spell checker for natural language processing in vaccine safety BMC Medical Informatics and Decision Making 2007 7 3 10.1186/1472-6947-7-3 PMC1805499 17295907

[B30] Mykowiecka A Marciniak M Domain driven automatic spelling correction for mammography reports Intelligent Information Processing and Web Mining 2006 5 521 530

[B31] Senger C Kalstschmidt J Schmitt SPW Pruszydlo MG Haefeli WE Misspellings in drug information system queries: characteristics of drug name spelling errors and strategies for their prevention International Journal of Medical Informatics 2010 79 12 832 839 10.1016/j.ijmedinf.2010.09.005 20951634

[B32] Meystre SM Savova GK Kipper-Schuler KC Hurdle JF Exracting information from textual documents in the electronic health record: a review of recent research Yearb Med Inform 2008 128 144 18660887

[B33] Uzuner Ö South BR Shen S Duvall SL 2010 i2b2/va challenge on concepts, assertions, and relations in clinical text Journal of the American Medical Informatics Association 2011 18 5 552 556 10.1136/amiajnl-2011-000203 21685143 PMC3168320

[B34] Uzuner Ö Solti I Cadag E Extracting medication from clinical text Journal of the American Medical Informatics Association 2010 17 5 514 518 10.1136/jamia.2010.003947 20819854 PMC2995677

[B35] Grouin C Deléger L Rosier A Temal L Dameron O Van Hille P Burgun A Zweigenbaum P Automatic computation of CHA2DS2-VASc score: information extraction from clinical texts for thromboembolism risk assessment AMIA Annual Symposium 2011 in press PMC3243195 22195104

